# Feasibility of Behavioral Intervention and Guided Stepping Training Early Post-Stroke (BIG STEPS): A Pilot Randomized Trial

**DOI:** 10.1016/j.arrct.2026.100665

**Published:** 2026-06-08

**Authors:** Victor E. Ezeugwu, Mona Iyizoba, Asya Shiloff-Rogers, Joy C. Ezeugwa, Sophia E. Pawelko, Andrew Chan, Jake Hayward, Carmen Tuchak, Jaime C. Yu

**Affiliations:** a Department of Physical Therapy, Faculty of Rehabilitation Medicine, University of Alberta, Edmonton, AB; b Glenrose Rehabilitation Hospital, Edmonton, AB; c Department of Emergency Medicine, Faculty of Medicine and Dentistry, University of Alberta, Edmonton, AB; d Division of Physical Medicine and Rehabilitation, Faculty of Medicine and Dentistry, University of Alberta, Edmonton, AB, Canada

**Keywords:** Assessment, Neurorehabilitation, Physical therapy, Rehabilitation, Stroke

## Abstract

**Objective:**

To evaluate the feasibility and preliminary effects of a behavioral intervention with guided stepping training early poststroke (BIG STEPS).

**Design:**

Pilot waitlist-controlled randomized trial.

**Setting:**

Community.

**Participants:**

Twenty-eight individuals with ischemic or hemorrhagic stroke (13 women [46%]; mean age: 67.8±13.3y).

**Interventions:**

A 12-week BIG STEPS intervention to reduce sedentary time and increase daily steps was delivered to the early BIG STEPS group (n=14) and, after a 12-week waitlist period, to the delayed BIG STEPS group (n=14). Both groups received usual care.

**Main Measures:**

Feasibility, adherence, and safety. Exploratory outcomes included accelerometer-derived movement behaviors, timed walking tests, and patient-reported outcomes.

**Results:**

Recruitment averaged 2.3 participants per month. Retention exceeded a priori threshold of 80% at weeks 12 and 24 (86%-93%). No intervention-related serious adverse events occurred. At week 12, the early BIG STEPS group accumulated a mean of 6534±2773 steps/day compared with 4031±2072 steps/day in the delayed BIG STEPS group. Sedentary time decreased by ∼1.4 h/d in the early group and 0.7 h/d in the delayed group. Between-group effects favored the early group for daily steps (Hedges’ *g*=1.4) and reduced sedentary time (Hedges’ *g*=−1.1). At week 24, outcomes converged after the delayed BIG STEPS group received the intervention. Between-group differences for timed walking tests and patient-reported outcomes were small.

**Conclusion:**

A behavioral intervention combined with guided stepping training early after stroke was feasible and safe, with promising preliminary effects.

Advances in acute stroke care have improved survival,^1^ yet many stroke survivors live longer with persistent functional limitations that restrict mobility, participation, and quality of life.^2^ Despite the well-established benefits of exercise in stroke rehabilitation,^3^, ^4^, ^5^ many stroke survivors remain insufficiently active after hospital discharge.^6^ Barriers include individual-level factors such as cardiovascular concerns,^7^ limited walking capacity,^8^ and low self-efficacy.^8^ As a result, physical activity during daily life after stroke remains low throughout the recovery period.

Daily step count is a practical, patient-centered indicator of real-world walking activity. In the general population, each additional 1000 steps/d is associated with ∼10%-15% lower risk of all-cause mortality,^9^ and accumulating about 7000 steps/d is associated with substantially reduced risk of cardiovascular disease and all-cause mortality.^10^^,^^11^ In stroke survivors, achieving ∼6000 steps/d has been associated with lower risk of recurrent vascular events.^12^ However, step counts commonly remain well below these thresholds after stroke, even in the chronic phase (≥6mo poststroke).^6^ This gap highlights an opportunity to target real-world walking recovery early after stroke using behavioral strategies that can be delivered alongside usual care.^13^ The early poststroke period represents a critical window for behavioral intervention, given heightened neuroplasticity during this period.^14^^,^^15^

Behavioral strategies such as goal setting, feedback, and self-monitoring, combined with guided progression of walking activity, may support sustainable changes in daily movement behaviors.^16^^,^^17^ However, stroke rehabilitation has traditionally prioritized improving functional capacity within structured settings, with less emphasis on translating gains into real-world behaviors after discharge. Building on prior work demonstrating the feasibility of a home-based sedentary behavior intervention after stroke,^18^ the Behavioral Intervention and Guided Stepping Training Early Post-Stroke (BIG STEPS) intervention integrates behavioral coaching with structured step-goal progression to increase daily steps and reduce sedentary time. The primary objective of this pilot trial was to evaluate the feasibility of BIG STEPS in individuals enrolled in the early subacute phase (<3mo) of stroke recovery. Secondary objectives were to estimate preliminary effects on daily step counts, activPAL-derived movement behaviors, timed walking tests, and patient-reported outcomes to inform a future definitive trial.

## Methods

### Trial design

A randomized waitlist-control pilot trial was conducted from June 2024 to November 2025. The study protocol was registered with ClinicalTrials.gov (NCT05753761). Before recruitment for this trial phase, a protocol amendment extended the intervention to 12 weeks, with assessments at baseline, week 12, and week 24. Participants allocated to the early BIG STEPS group received the intervention immediately following baseline measurements, in addition to usual poststroke care. Participants allocated to the delayed (waitlist) group received usual care for 12 weeks before crossing over to receive the BIG STEPS intervention. Outcome assessors were blinded to group allocation. Blinding of participants and the interventionist was not possible, given the nature of the intervention. Trial reporting followed the CONSORT 2010 extension for randomized pilot and feasibility trials,^19^ and the 2025 CONSORT checklist (supplemental table S1).^20^ Ethics approval was granted by the Institutional Health Research Ethics Board (Pro00140017), and all participants provided written informed consent.

### Participants

Screening and recruitment of participants took place between June 2024 and May 2025. Participants who were returning home or had recently returned home from the hospital were recruited through an inpatient stroke program, a specialized rehabilitation outpatient program, and a stroke early supported discharge program. Eligible participants were: adults (≥18 years of age) with ischemic or hemorrhagic stroke within 3 months since onset, capable of walking independently or with supervision for ≥5 meters, with or without a gait aid. We excluded participants with previously diagnosed mobility-limiting neurological conditions, severe aphasia, or cognitive impairments that precluded the ability to understand or follow instructions.

### Procedure

Two physical therapists introduced the study to potential participants. After obtaining written informed consent, demographic and clinical information were collected via chart review, including age, sex, height, weight, marital status, days since stroke onset, stroke type, stroke severity at onset, lipid profile at onset, and inpatient rehabilitation length of stay. At each assessment time point, participants wore an activPAL sensor (PAL Technologies)^a^ for 4 consecutive days to measure 24-hour movement behaviors. The activPAL^a^ is a thigh-worn triaxial accelerometer and inclinometer that classifies posture and movement (sitting/lying, standing, and stepping) based on acceleration and thigh inclination and quantifies step count, sedentary time, and upright time.^21^ The monitor was secured to the midpoint of the anterior nonhemiparetic thigh (or the right thigh if no weaker side) using a Tegaderm film dressing (Solventum).^b^ At least 2 valid days yields reliable estimates of movement behaviors after stroke.^22^ Data were downloaded and processed using PAL software (PAL Technologies),^a^ applying the CREAtive/updated PAL software algorithm (CREA) classification algorithm.

**Fig 1:**
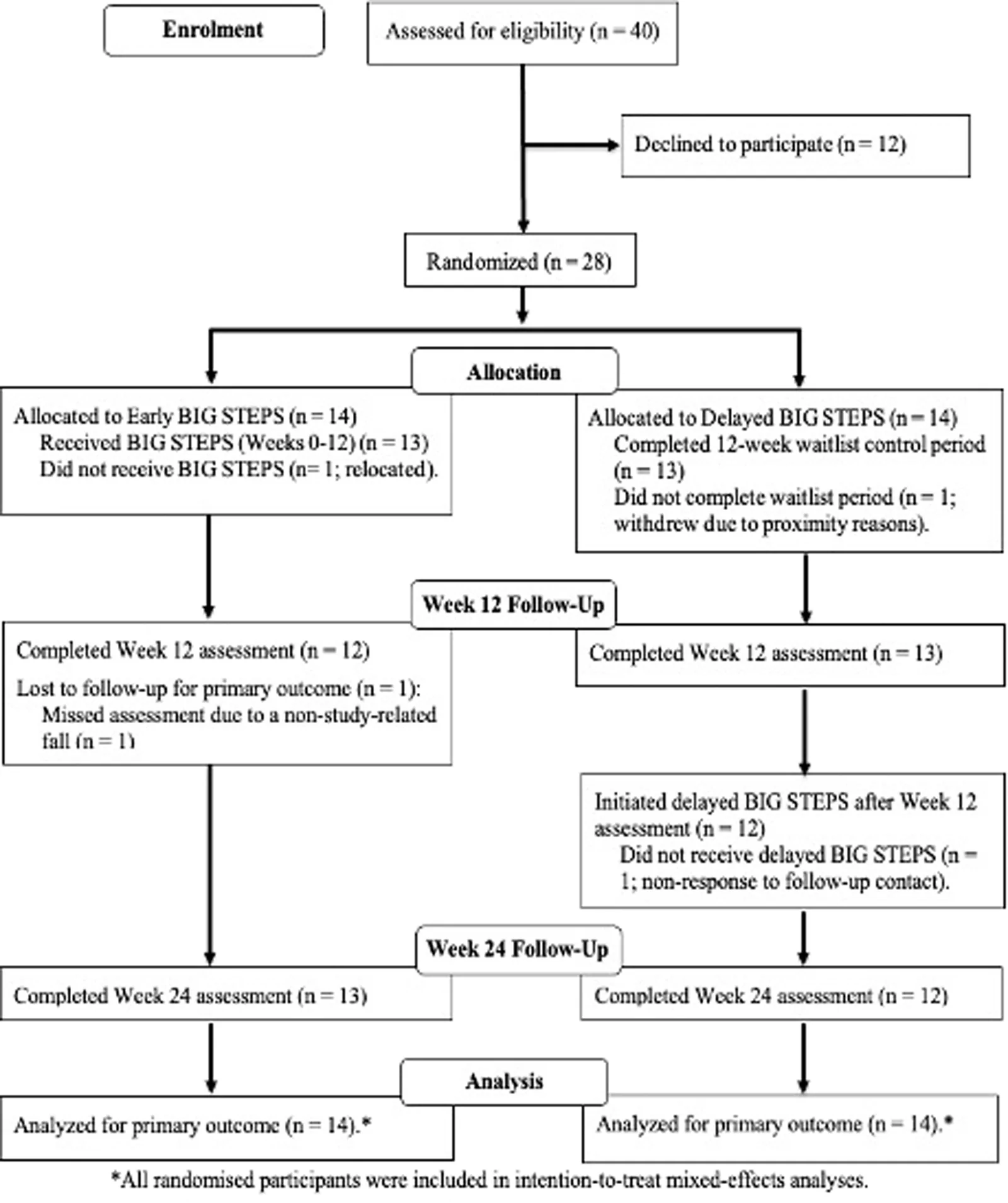
CONSORT patient flow diagram

Following baseline assessment, eligible participants were randomized 1:1 to either the early BIG STEPS intervention group or the delayed BIG STEPS group. The allocation sequence was generated by a study team member not involved in recruitment or data collection using an online randomization tool (studyrandomizer.com) with permuted block sizes of 2-4. Randomization was stratified by stroke program (inpatient rehabilitation, specialized rehabilitation outpatient program, and stroke early supported discharge program) to ensure balanced representation across strata. The flow of participants through the study is presented in figure 1.

### Interventions

All participants received usual poststroke care, which typically included mobility, balance, and strengthening exercises provided by physical therapists and physical therapy assistants. BIG STEPS was a 12-week behavioral intervention adapted from a previously published protocol.^23^ Based on social cognitive theory,^24^ the intervention was designed to reduce and frequently interrupt sedentary time while progressively increasing daily step counts. Participants received a minimum of 1 in-person home session delivered by a licensed physical therapist, followed by 11 virtual sessions; additional home-visits were provided as needed to troubleshoot self-monitoring device malfunctions. Participants performed “sit-less, move-more” activities independently at home and received an intervention handbook and a reference table of age- and sex-stratified normative steps/day.^25^ Baseline activPAL^a^ data were used to establish individualized initial step goals, and participants used a Fitbit Inspire 2 or 3 (Fitbit)^c^ activity tracker for self-monitoring. To protect privacy, a study-specific account was created for each participant. The research team used Fitbit data to monitor adherence and progressively adjust weekly step goals based on the previous week’s performance.^26^, ^27^, ^28^

### Primary outcome

The primary outcome of this pilot trial was feasibility, including recruitment, retention, adherence, and safety. Recruitment rate was defined as the number of participants enrolled per month. Retention was defined as the proportion of enrolled participants who completed outcome assessments at weeks 12 and 24. Adherence was defined as engagement with the intervention and compliance with monitoring procedures. Device adherence was assessed as the proportion of participants providing activPAL^a^ data for ≥2 valid wear days per assessment period (a valid day defined as 24h of continuous recording) and the number of days with recorded Fitbit step data during the intervention period. Participant safety was monitored weekly through verbal questioning and documentation of reported adverse events in electronic medical records. A priori feasibility and progression criteria were established to guide interpretation of trial outcomes and inform readiness to progress to a definitive trial (table 1). Feasibility was interpreted using a predefined traffic light progression system^29^; red (stop) if the recruitment criterion was not met; amber (modify) if recruitment was met and at least one of the remaining criteria reached ≥70% but did not meet the pre-specified benchmark; and green (proceed) if all progression criteria were met. Meeting all criteria was considered indicative of suitability to progress to a definitive trial.

**Table 1:** A priori feasibility and progression criteria for the BIG STEPS pilot randomized trial

Domain	Criterion
Recruitment	1-2 participants recruited per month.
Retention	≥80% of participants complete outcome assessments at 12 and 24 wk.
Adherence	≥80% of participants attend all intervention sessions, provide valid activPAL data for ≥2 d at each assessment time point, and Fitbit step data on ≥70 of 84 intervention days.
Safety	No serious intervention-related adverse events and no systematic pattern of adverse events related to study participation.

### Exploratory outcomes

Exploratory outcomes were assessed at each time point and included activPAL-derived movement behaviors, timed walking measures, and patient-reported outcomes. Timed walking assessments included walking endurance using the 6-minute walk test (6MWT),^30^ timed Up and Go (TUG) test,^31^ and comfortable and fastest gait speeds.^30^ Patient-reported outcomes included Patient Health Questionnaire-9,^32^ Fatigue Severity Scale,^33^ and EuroQol 5-Dimension visual analog scale (EQ-5D VAS).^34^

Cohort descriptors at stroke onset extracted from medical records included the National Institutes of Health Stroke Scale^35^ and vascular risk markers–glycated hemoglobin (HbA1c), low-density lipoprotein cholesterol, and triglycerides.

### Data analysis

Baseline participant characteristics were summarized using descriptive statistics, and feasibility outcomes were reported using frequencies and percentages. Exploratory outcomes were analyzed using linear mixed-effects models fitted with restricted maximum likelihood estimation. Models included fixed effects for group, time point, and group-by-time interaction, with random intercepts for participants.^36^ Adjusted between-group mean differences at weeks 12 and 24 were derived from model-based estimated marginal means and are presented with 95% confidence interval (CI). Standardized effect sizes (Hedges’ g) were calculated by dividing the adjusted between-group mean difference by the pooled SD of the observed baseline values, with a small-sample correction applied. Effect sizes of ∼0.2, 0.5, and 0.8 were interpreted as small, medium, and large, respectively. Hedges’ *g* was selected as it provides a less biased estimate than Cohen’s d in small samples. Mixed-effects models included all available observations under the missing-at-random assumption, and no imputation was performed.^36^ All analyses were conducted using Stata version 18^d^ (StataCorp).

## Results

### Participants

Participant characteristics are presented in table 2. The mean age was 67.8±13.3 years, and 46% (13/28) of participants were women. Participants were enrolled a mean of 49.1±19.4 days after stroke, and most had ischemic stroke (82%). Stroke severity was mild to moderate (National Institutes of Health Stroke Scale: 6.3±4.5). The mean body mass index was 26.5±4.0 kg/m², and 57% of participants lived with a partner. Participants had a mean inpatient rehabilitation length of stay of 27.8±10.4 days.

**Table 2:** Baseline characteristics by treatment group

	All (n=28)	Early BIG STEPS (n=14)	Delayed BIG STEPS (n=14)
Age (y)	67.8±13.3	72.3±10.6	63.3±14.5
Sex (n, % women)	13 (46.4)	7 (53.9)	6 (46.2)
Body mass index (kg/m^2^)	26.5±4.0	25.3±4.0	27.7±3.8
Lives with a partner (n, % yes)	16 (57.1)	8 (50.0)	8 (50.0)
Time since stroke (d)	49.1±19.4	49.2±22.7	48.9±16.4
Type of stroke (n, % ischemic)	23 (82.1)	11 (78.6)	12 (85.7)
Stroke location			
Subcortical	10 (35.7)	6 (42.9)	4 (28.6)
Posterior circulation	8 (28.6)	4 (28.6)	4 (28.6)
MCA territory	7 (25.0)	3 (21.4)	4 (28.6)
Mixed	3 (10.7)	1 (7.1)	2 (14.3)
Stroke severity at onset (NIHSS)	6.3±4.5	5.2±4.6	7.3±4.4
HgbA1c at stroke onset	6.2±1.2	6.6±1.6	5.9±0.6
Low-density lipoprotein	2.6±1.2	2.5±1.4	2.8±1.1
Triglycerides	1.4±0.7	1.5±0.8	1.3±0.6
Length of inpatient rehabilitation (d)	27.8±10.4	25.7±10.4	29.6±10.4

NOTE. Values are mean±SD or n (%). Stroke location: subcortical includes thalamus, basal ganglia, internal capsule, and lentiform nucleus; posterior circulation includes posterior cerebral artery, cerebellum, and brainstem; mixed includes multifocal or multi-territory strokes.

Abbreviations: HgbA1c, hemoglobin A1c; MCA, middle cerebral artery; NIHSS, National Institutes of Health Stroke Scale.

### Usual care

Participants in the early and delayed BIG STEPS groups continued to receive usual-care physical therapy during the study period. Between baseline and week 12, participants in the early BIG STEPS group completed a median of 4 physical therapy sessions (interquartile range [IQR], 1-9; range, 0-17), whereas the delayed BIG STEPS group completed 11 sessions (IQR, 5-13; range, 1-18). After crossover at 12 weeks, participants in the delayed BIG STEPS group completed a median of 1 physical therapy session (IQR, 0-14; range, 0-18) between weeks 12 and 24.

### Feasibility

#### Recruitment

Between June 2024 and May 2025, 28 participants were enrolled, yielding an average recruitment rate of 2.3 participants per month.

#### Retention

As shown in figure 1, all participants (n=28) completed baseline assessments. At week 12, 12 of 14 participants (86%) in the early BIG STEPS group and 13 of 14 participants (93%) in the delayed BIG STEPS group completed outcome assessments.

#### Adherence

**Fig 2:**
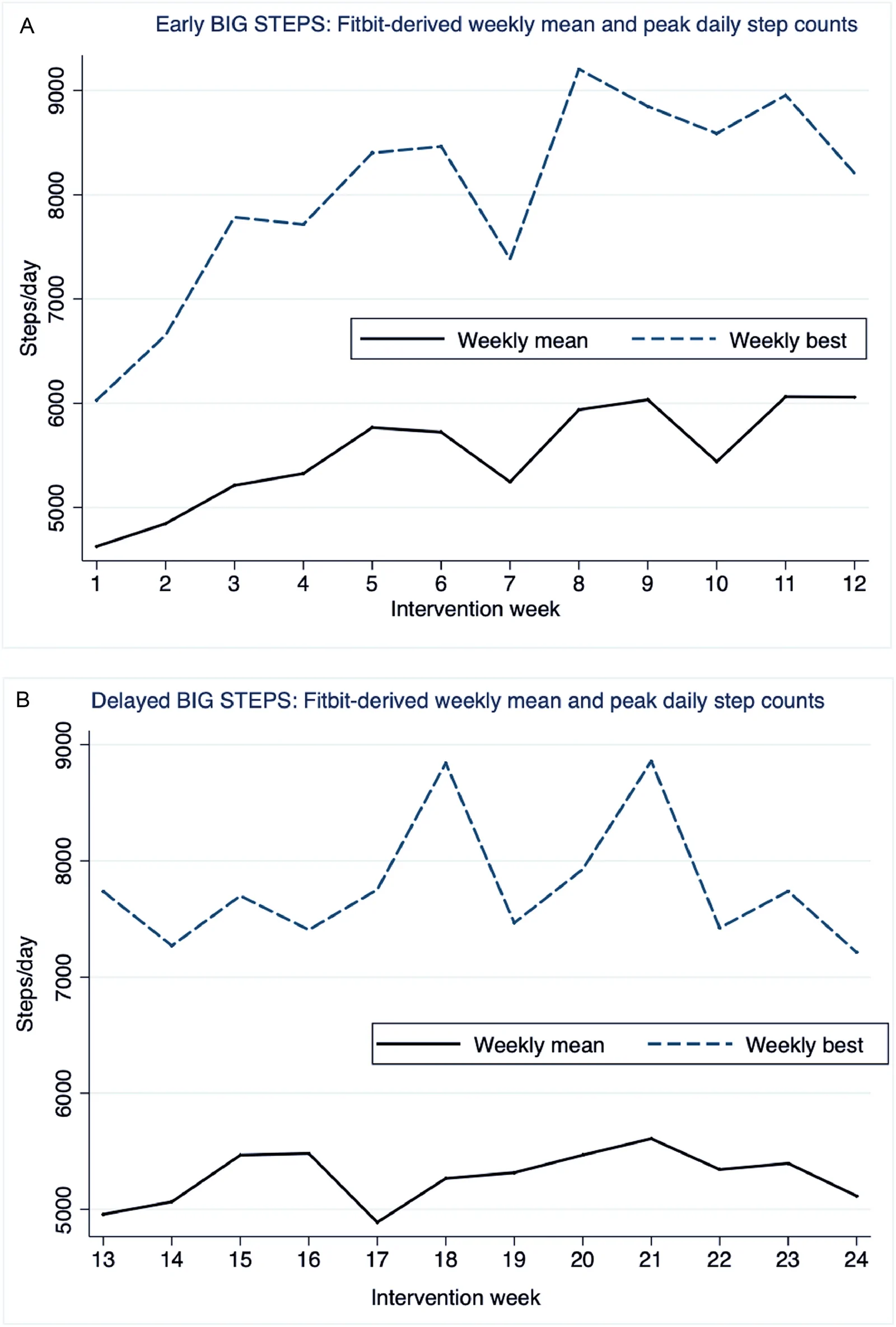
(A) Weekly mean and peak Fitbit steps/day for early BIG STEPS; (B) weekly mean and peak Fitbit steps/day for delayed BIG STEPS

Intervention adherence was high. During the 12-week intervention period, all scheduled intervention sessions were attended. The mean number of days with recorded Fitbit^c^ step data was 80.5±6.7 days in the early BIG STEPS group and 80.8±8.9 days in the delayed BIG STEPS group, corresponding to ∼96% Fitbit^c^ data completeness and exceeding predefined adherence benchmarks. Two participants wore the Fitbit^c^ on the ankle using an ankle band because of low accelerations when holding onto walking aids. Figures 2A and B illustrates trajectories of Fitbit-derived weekly average daily steps and best weekly performance during the intervention periods. The early BIG STEPS group demonstrated slightly higher weekly averages and a more consistent increasing trend, reaching 6000 steps/d at weeks 9, 11, and 12.

#### Safety

No serious adverse events attributable to the BIG STEPS intervention were reported. At baseline, 1 participant in each group reported a fall. At week 12, 2 participants in each group reported a fall; one participant in the early BIG STEPS group sustained a humeral fracture after falling while doing laundry. At week 24, 1 participant in each group reported a fall. Overall, fall rates were comparable between groups and did not appear to be intervention related.

### Exploratory outcomes

#### Accelerometry

Descriptive accelerometry outcomes are presented in table 3, with adjusted between-group differences shown in table 4. At the week-12 primary endpoint, the early BIG STEPS group accumulated 6534±2773 steps/d, compared with 4031±2072 steps/d in the delayed BIG STEPS group. Sedentary time was also lower in the early BIG STEPS group by ∼106 min/d. Correspondingly, large between-group effects were observed for daily step counts (Hedges’ *g*=1.4) and sedentary time (Hedges’ *g*=−1.1). Within-group changes are summarized in supplemental table S2. From baseline to week 12, the early BIG STEPS group increased daily step counts by 1911 steps/d (95% CI, 840-2981) and reduced sedentary time by 86.6 min/d (95% CI, −144.2 to −29.0). In contrast, the delayed BIG STEPS group showed smaller changes during the waitlist period. As expected, between-group differences were attenuated at week 24 after both groups had received BIG STEPS.

**Table 3:** Exploratory outcomes at baseline, 12 weeks, and 24 weeks

	Early BIG STEPS (mean±SD)			Delayed BIG STEPS (mean±SD)		
Outcomes	Week 0	Week 12	Week 24	Week 0	Week 12*	Week 24*
Accelerometry						
Steps (n)	4598±1545	6534±2773	5994±2118	3016±2028	4031±2072	4638±1947
Stepping time (min)	64.7±17.8	87.8±32.0	78.9±23.9	44.9±26.8	60.4±29.2	66.1±26.5
Standing time (min)	185.2±94.9	221.6±57.9	238.6±95.0	129.0±65.2	176.9±53.1	173.9±49.9
Sedentary time (min)	669.9±112.9	580.4±96.9	626.9±114.8	733.6±64.1	687.5±129.2	685.4±92.1
Primary lying time (min)	519.7±107.4	547.7±78.6	487.8±98.1	530.1±98.1	512.7±144.1	512.3±117.1
Timed walking measures						
6MWT distance (m)	351.4±83.1	360.5±68.4	364.0±92.4	294.3±124.2	355.3±122.1	379.2±111.7
TUG (s)	14.5±4.2	10.8±2.8	13.7±11.1	20.2±11.7	13.0±5.0	10.7±3.2
CGS (m/s)	1.0±0.2	1.0±0.2	1.1±0.2	0.8±0.2	0.9±0.3	1.0±0.2
FGS (m/s)	1.3±0.3	1.3±0.3	1.4±0.3	1.0±0.4	1.2±0.4	1.3±0.3
Patient-reported outcomes						
PHQ-9 (0-27)	5.2±3.7	4.9±3.0	3.3±3.4	4.1±4.4	6.2±4.3	6.0±5.0
FSS (9-63)	32.9±10.6	33.7±12.2	28.3±13.7	25.9±10.7	33.2±12.9	38.4±10.5
EQ-5D VAS (0-100)	65.0±19.0	75.4±13.4	73.9±16.3	62.9±17.8	68.3±17.5	70.5±15.2

NOTE. *Week 12 represents the end of the waitlist period; week 24 represents completion of the delayed BIG STEPS intervention.

Abbreviations: CGS, comfortable gait speed; FGS, fastest gait speed; FSS, fatigue severity scale; PHQ-9, 9-item Patient Health Questionnaire.

**Table 4:** Adjusted between-group mean differences in exploratory outcomes at 12 and 24 weeks

	Early Delayed BIG STEPS at 12 weeks			Early Delayed BIG STEPS at 24 weeks		
Outcomes	MD	95% CI	Hedges’ *g*	MD	95% CI	Hedges’ *g*
Accelerometry (per day)						
Steps (number)	2583	978-4188	1.4	1576	−29 to 3181	0.9
Stepping time (min)	27.5	7.3-47.7	1.2	15.2	−5.0 to 35.4	0.7
Standing time (min)	64.1	5.8-122.4	0.8	66.5	8.2-124.7	0.8
Sedentary time (min)	−106.2	−186.6 to −25.8	−1.1	−59.6	−140.0 to 20.8	−0.6
Primary lying time (min)	15.9	−68.0 to 99.9	0.2	−28.1	−112.0 to 55.8	−0.3
Timed walking measures						
6MWT distance (m)	17.8	−60.8 to 96.3	0.2	10.2	−69.6 to 90.0	0.1
TUG (sec)	−1.8	−7.7 to 4.1	−0.2	2.6	−3.6 to 8.8	0.3
CGS (m/sec)	0.2	−0.1 to 0.4	0.5	0.2	−0.1 to 0.4	0.5
FGS (m/sec)	0.1	−0.1 to 0.3	0.6	0.1	0-0.3	0.6
Patient-reported outcomes						
PHQ-9 (0-27)	−0.9	−3.9 to 2.2	−0.2	−2.1	−5.3 to 1.1	−0.5
FSS (9-63)	0.8	−8.4 to 10.1	0.1	−9.9	−19.7 to 0	−0.9
EQ-5D VAS (0-100)	5.5	−7.4 to 18.3	0.3	0.7	−12.7 to 14.1	0

NOTE. The delayed group received BIG STEPS intervention between weeks 12 and 24.

CGS, comfortable gait speed; FGS, fastest gait speed; FSS, fatigue severity scale; MD, mean difference; PHQ-9: 9-item Patient Health Questionnaire.

#### Timed walking tests

Timed walking test results, including walking endurance (6MWT), functional mobility (TUG), and gait speed, are summarized in table 3, with adjusted between-group differences presented in table 4. At week 12, between-group effects (Hedges’ *g*=−0.3 to 0.7) were small to modest for walking endurance and gait speed, with negligible effects for TUG. Within-group changes are presented in supplemental table S2. From baseline to week 12, the delayed BIG STEPS group increased 6MWT distance by 47.8 m (95% CI, 17.5-78.0), whereas changes in the early BIG STEPS group over the same period were smaller. After crossover at week 12, the delayed BIG STEPS group demonstrated further gains, with 6MWT distance increasing to 65.7 m (95% CI, 32.2-99.1) from baseline to week 24. Across both groups, improvements over time were observed in functional mobility (TUG), with smaller, consistent gains in gait speed.

#### Patient-reported outcomes

Patient-reported outcomes are summarized in table 3, with adjusted between-group comparisons shown in table 4, and within-group changes reported in supplemental table S2. At week 12, between-group differences were small across outcomes. Fatigue severity tended to increase during the waitlist period in the delayed BIG STEPS group and was lower in the early BIG STEPS group by week 24. Changes in depressive symptoms were minimal across time points. Health-related quality of life (EQ-5D VAS) was modestly higher following delivery of the intervention in both groups.

## Discussion

This pilot randomized waitlist-controlled trial evaluated the feasibility, safety, and preliminary effects of BIG STEPS delivered during the early recovery period following stroke. Feasibility outcomes met all predefined progression criteria for a definitive trial, demonstrating adequate recruitment, high retention, excellent adherence, and no serious adverse events attributable to the intervention. Intervention features such as self-monitoring and weekly follow-ups likely contributed to the high adherence and the observed effects. Mastery of self-monitoring can strengthen self-efficacy, a key component of the BIG STEPS intervention.^37^^,^^38^ In a previous study,^38^ participants reported that self-monitoring was a strong source of motivation and that achieving daily step goals provided personal gratification.^38^ Once engaged with the intervention, participants often described forming a habit of getting up to move at regular intervals, even without consciously checking their monitors.^38^ The benefit of regular follow-ups after hospital discharge is also important. Once discharged from the hospital, the programs that stroke survivors can access to help maintain activity and function are often less structured or less available, highlighting the importance of interventions such as BIG STEPS.^39^^,^^40^ In addition, relatively few studies have investigated the use of activity monitors to increase daily steps or reduce sedentary time after stroke,^41^^,^^42^ although evidence suggests that wearable trackers are most effective when paired with behavior change components such as goal setting and health coaching.^43^

Exploratory analyses showed large between-group effects at the 12-week primary endpoint for daily step counts (Hedges’ *g*=1.4) and sedentary time (*g*=−1.1), with expected attenuation of between-group differences after crossover when the delayed BIG STEPS group received the intervention. Improvements in daily step counts and reductions in sedentary time observed with the BIG STEPS intervention are consistent with evidence showing that combined interventions of health coaching and progressive goal setting can increase free-living walking activity after stroke.^27^^,^^44^^,^^45^ In the PROWALKS trial, gains in steps/day were observed following step activity monitoring and progressive goal setting after stroke, supporting the importance of behavior change strategies for shifting daily activity patterns.^27^ Our findings extend emerging stroke research showing that behavior change approaches can feasibly and safely reduce sedentary behavior after stroke.^18^^,^^42^^,^^46^^,^^47^ These studies support combining objective monitoring, individualized goal progression, and therapist support to improve outcomes after stroke. Delivering BIG STEPS during the subacute phase and shortly after transition from hospital to home is a novel aspect of this trial, targeting a critical period of stroke recovery often characterized by reduced access to rehabilitation services.

Between-group effects for timed walking tests were modest (Hedges’ *g*=−0.2 to 0.6), with small mean differences in 6MWT distance (17.8m at week 12; 10.2m at week 24) and CIs crossing zero. These findings highlight the distinction between performance (what individuals do in daily life) and capacity (what they can do under test conditions).^48^ This behavioral specificity is consistent with the intervention’s theoretical framework.^24^^,^^49^ The greater improvement in 6MWT distance observed in the delayed BIG STEPS group during the waitlist period may partly reflect higher exposure to usual-care physical therapy, which targets walking capacity (median 11 sessions [IQR, 5-13] vs 4 sessions [IQR, 1-9] in the early BIG STEPS group).

Patient-reported outcomes demonstrated a more variable pattern. Health-related quality of life (EQ-5D VAS) improved modestly over time in both groups. Depressive symptoms (Patient Health Questionnaire-9) improved in the early intervention group but worsened during the waitlist period in the delayed BIG STEPS group, suggesting a potential benefit of earlier engagement with the intervention. In contrast, fatigue showed a less consistent trajectory, particularly in the delayed intervention group, where scores increased over time, including after receipt of the intervention. This pattern aligns with evidence that poststroke fatigue is a common and persistent concern that may act as a barrier to recovery.^50^ It is possible that increasing daily steps may transiently increase perceived fatigue as individuals adapt to higher activity levels. Fatigue after stroke is heterogeneous and often managed through self-developed strategies such as pacing, activity planning, and education.^51^ In the early intervention group, fatigue remained relatively stable immediately after the intervention and improved at follow-up, which may reflect this adaptation process.

Strengths of this study include the adaptive, individually tailored behavioral intervention with guided stepping training, objective monitoring to assess intervention fidelity, and achievement of predefined feasibility metrics. Study Limitations: This study also has several limitations. Compared with the originally published protocol,^23^ this pilot study recruited participants within 3 months of stroke onset and delivered a 12-week intervention. These adjustments were made to include participants with both ischemic and hemorrhagic stroke, as the safety of BIG STEPS within 7 days of hemorrhagic stroke was uncertain during the original study design. Cognition was assessed during routine predischarge care; all BIG STEPS participants were cleared to return home, and no additional screening was conducted. Although engagement with the intervention was excellent, acceptability was inferred from adherence and retention rather than assessed using structured qualitative methods.

## Conclusion

In conclusion, BIG STEPS was feasible and safe in the subacute phase of stroke recovery. The large effects observed in daily step counts and sedentary time at the primary endpoint, combined with strong feasibility metrics, support progression to a fully powered randomized controlled trial. A definitive trial with an optimized design, including extended follow-up to 24 weeks before the delayed group receives BIG STEPS, is required to determine whether early improvements translate into sustained changes in movement behaviors.

## Suppliers

a. activPAL, PAL software; PAL Technologies Ltd.
b. Tegaderm film dressing; Solventum.
c. Fitbit Inspire 2 and 3; Fitbit.
d. Stata, version 18.0; StataCorp.

## Disclosure

The investigators have no financial or nonfinancial disclosures to make in relation to this project.

## Appendix Supplementary materials

Image, application 1

Image, application 2

## Footnotes

Supplementary material associated with this article can be found, in the online version, at doi:10.1016/j.arrct.2026.100665.
