# Supplementary material for: Feasibility of Behavioral Intervention and Guided Stepping Training Early Post-Stroke (BIG STEPS): A Pilot Randomized Trial
Source: Arch Rehabil Res Clin Transl. 2026 Jun 8;8(3):100665. doi: 10.1016/j.arrct.2026.100665 (PMC13591194; doi:10.1016/j.arrct.2026.100665)
Supplement: Supplementary file 2 [file mmc2.docx]

**Supplementary Table 2**. Within-group change from baseline to 12 and 24 weeks for exploratory outcomes

| Outcomes | Early BIG STEPS (mean change (95% CI) | |  | Delayed BIG STEPS (mean change (95% CI) | |
| --- | --- | --- | --- | --- | --- |
|  | 0-12 weeks | 0-24 weeks |  | 0-12 weeks | 0-24 weeks |
| Accelerometry (per day) |  |  |  |  |  |
| Steps (number) | 1911 (840 to 2981) | 1381 (340 to 2421) |  | 910 (-131 to 1951) | 1387 (317 to 2457) |
| Stepping time (minutes) | 22.0 (7.5 to 36.6) | 13.9 (-0.3 to 28.0) |  | 14.4 (0.3 to 28.5) | 18.5 (4.0 to 33.0) |
| Standing time (minutes) | 51.5 (20.2 to 82.9) | 50.1 (19.6 to 80.6) |  | 43.6 (13.1 to 74.1) | 39.8 (8.4 to 71.2) |
| Sedentary time (minutes) | -86.6 (-144.2 to -29.0) | -36.5 (-92.5 to 19.6) |  | -44.1 (-100.1 to 12.0) | -40.5 (-98.1 to 17.1) |
| Primary lying time (minutes) | 11.6 (-41.3 to 64.6) | -36.9 (-88.3 to 14.6) |  | -14.8 (-66.2 to 36.7) | -19.2 (-72.2 to 33.7) |
| Timed walking measures |  |  |  |  |  |
| 6MWT distance (meters) | 8.4 (-22.8 to 39.6) | 18.8 (-12.4 to 49.9) |  | 47.8 (17.5 to 78.0) | 65.7 (32.2 to 99.1) |
| TUG (seconds) | -3.5 (-6.9 to 0) | -3.3 (-6.8 to 0.3) |  | -6.5 (-9.9 to -3.2) | -8.9 (-12.6 to -5.2) |
| CGS (metres/second) | 0.1 (0 to 0.1) | 0.1 (0 to 0.2) |  | 0.1 (0 to 0.2) | 0.1 (0.1 to 0.2) |
| FGS (metres/second) | 0 (-0.1 to 0.1) | 0.1 (0 to 0.2) |  | 0.1 (0 to 0.2) | 0.2 (0.1 to 0.3) |
| Patient-reported outcomes |  |  |  |  |  |
| PHQ-9 (0-27) | -0.1 (-2.2 to 1.9) | -1.6 (-3.7 to 0.4) |  | 1.8 (-0.2 to 3.8) | 1.5 (-0.7 to 3.7) |
| FSS (9-63) | 0.9 (-7.3 to 9.2) | -4.3 (-12.5 to 4.0) |  | 7.1 (-1.0 to 15.1) | 12.5 (3.8 to 21.3) |
| EQ-5D VAS (0-100) | 8.9 (0.4 to 17.4) | 8.6 (0.1 to 17.2) |  | 5.6 (-2.8 to 13.9) | 10.0 (0.9 to 19.1) |

*Note*: Values represent model-estimated mean change from baseline (95% CI) derived from linear mixed-effects models. Week 12 represents completion of the initial intervention phase for Early BIG STEPS and the end of the waitlist period for the Delayed group. Week 24 represents completion of the delayed BIG STEPS intervention.
